# A Treatise on Midwifery; Developing New Principles, Which Tend Materially to Lessen the Sufferings of the Patient, and Shorten the Duration of Labour

**Published:** 1819-10-01

**Authors:** 


*A Treatise on Midwifery; developing New Principles, which tend materially to lessen the Sufferings of the Patient, and shorten the Duration of Labour.* By JOHN POWER, Accoucheur, &c. One Vol. Octavo, pp. 270. London, 1819.

In no process or operation of the animal economy are the wonderful powers of NATURE more fully manifested than in parturition. A celebrated physiologist and accoucheur, in this Metropolis, used to remark in his lectures, that the said process was even "*beautiful to behold.*" For our own parts, while we admired the means and resources of Nature, in the parturient efforts, we were quite content with a *tangible* demonstration of them, leaving their optical attractions for connoisseurs of another order. But it is not in parturition alone that the great power of Nature is cognizable; and there is strong reason to believe that the apparent perfection of her operations has sometimes led us to a devotion that has blinded us to her defects; her occasional incapacities; nay, her irregular and even insalutary aberrations. Nor do we accuse *her* of these imperfections. We first desert Nature's standards; then turn a deaf ear to her calls, or run counter to her laws; and lastly, when we come to suffer the penalty of our follies, we expect that from Nature which we have not left her the power to perform. If we compare animals in a state of nature with those which are domesticated and effeminated under man, we find the female bring forth with greater difficulty and danger among the latter than among the former class. And if we institute a similar comparison between the woman of Otaheite and the lady of fashion in London, the contrast is still more striking.

It must always be borne in mind, that human parturition is more laborious, difficult, and dangerous, in civilized, than in uncivilized life, independently of those mechanical obstructions and morbid organizations which result from the former state of society. To what can this be owing but to the morbid sensibility, physical and moral, of the female system? It is to this very point that Mr. Power's investigations have been directed; and whatever our obstetrical brethren may say or think to the contrary, we are of opinion that our author has brought forward many ingenious views, and suggested many useful measures relative to the principles and practice of his art.

We cannot help thinking, indeed, that Mr. Power would have effected his purpose better by compressing the subject into a paper in one of the Journals or Transactions of the day, than by incorporating his original observations with a professed Treatise on Midwifery. We shall endeavour, however, to present the reader with the principal points of novelty and utility in this volume, without entering into the general principles or minute particulars of this branch of medical science.

In the human female, as well as in the animal, and even in the vegetable, a gradual state of *preparation* for the parturient effort is observable. In the earlier period of utero-gestation the cervix uteri sustains little alteration; but from the middle of pregnancy it begins to undergo a gradual series of expansions, until it is at length entirely obliterated, when the uterine contents come into immediate contact with the circle of the os tincæ, and then the whole apparatus is ready for the expulsive actions of the uterus.

For a few days previous to the commencement of the parturient process a subsidence of the abdominal uterine tumour becomes evident. This subsidence, our author concludes, is not referrible to diminution of substance, but arises from the uterus (which, during the period of utero-gestation, has never closely embraced its contents, but has surrounded them in a loose and relaxed manner) contracting itself upon them, as if ready and eager to commence the parturient process. This state of contraction, or increased tonicity, is most probably, thinks our author, owing to a concentration of nervous energy round the uterine system, to supply the great effort about to be made.

One probable effect of this uterine contraction may be to complete the expansion of the cervix uteri by the increased pressure from above. Examination, per vaginam, at this time will shew the head of the child applied to the circle of the os uteri, or, in case of obliquity, lying upon some lateral part of the cervix, producing a protrusion of it upon the vagina, and forming what has been called the uterine tumour.

A state of pain commonly precedes the expulsive action, which has been called spurious pain, and although it produces no dilating effect upon the os uteri, it is often so violent as to simulate true labour pains. These false pains occupy the belly, back, hips, or any other muscular parts adjoining the organs of parturition; and, at times, the uterus itself: they even produce sensations of fæcal and urinary evacuations, and are connected with various sympathetic actions throughout the system.

The fœtus having arrived at perfection, a series of parturient paroxysms commence for its expulsion. During the continuance of each, the patient is sensible of something pressing within her, which she compares to a sense of bearing down, resembling the nisus at the commencement of fæcal evacuation.

"On applying the hand to the abdomen of the patient, previous to the approach of the paroxysm, the uterus will be found in a flaccid state; the parts of the child and other abdominal contents may, indeed, be felt presenting harder masses through its parietes, but its general feel will offer an easy compressibility. The paroxysm now commences; it immediately becomes evident, even before the patient has a preconception of it, that a change is taking place. The compressibility gradually diminishes, until it is entirely lost, and the abdominal tumour is rendered so hard and tense, that a difficulty is found in producing the slightest indentation, so that it may be said, to speak emphatically, to become hard and solid as a board. As the paroxysm recedes, the contraction and incompressibility gradually go off, and the softness and flaccidity return." P. 24.

We cannot help thinking that the foregoing train of phenomena ought to be kept steadily in mind by the obstetric practitioner, since the state of uterine contraction, as evinced through the abdominal parietes, affords a very delicate and excellent mode of trying a pain, while the information thence derived is, our author asserts, most important and uniformly correct.

"No genuine parturient action is without it, and, when perfect, no false or unprofitable action is ever found coexistent with it, its presence evidencing the existence, and its absence the want of the true energetic uterine principle; by which it unfortunately happens that the most distressing states of parturition are often, for a length of time, totally unaccompanied." P. 25.

By attending to the above, we avoid the necessity of frequent examination *per vaginam,* so distressing to female delicacy, and often so painful to the corporeal feelings. The information may be obtained, too, through the dress of the patient, without trouble, or much appearance of interference. When *real* uterine paroxysms come on, however, the *progress* of the case can only be ascertained per vaginam.

In the fourth section of the work, Mr. Power discusses the *nature and causes* of the parturient action, and is very diffuse. After much analogical reasoning he comes to the conclusion that the contractions of the uterus are produced, in consequence of a certain impression excited by its contents upon its own orifice. This orifice, he avers, possesses a high degree of sensibility or nervous power, and is little connected with utero-gestation until that function is complete, being previously removed to a determinate distance from the distending process. This hypothesis, he thinks, is strengthened by the fact that, in wrong presentation, or malformation of the child or pelvis, or where the liquor amnii is excessive, and consequently where the more firm parts of the child cannot be applied to the uterine orifice, the parturient process is less forcibly or speedily excited. And again, the contractions of the os uteri may be artificially excited by an irritation applied to the orifice, affording a proof that the cause presumed is adequate to produce the effect attributed to it. This doctrine is also illustrated by a curious case, related by Mr. Power's father, where, in consequence of a very pendulous abdomen, a woman went nearly three months after the full period of utero-gestation. By means of a bandage the child's head was removed from its pendulous situation over the os pubis, and, in four or five days, labour pains came on.

In the chapter on the pathology of parturition, we observe a great many useful and ingenious hints that prove Mr. Power to be a man of close discernment. Our author now comes to a very interesting subject; namely, the metastasis or diversion of the real parturient effort from the uterus to neighbouring parts, the pain and suffering being great, but the profit nothing. Frequent opportunities are afforded of ascertaining, by application of the hand to the abdomen, even under the most acute states of painful paroxysm, that the uterus will continue, during that paroxysm, in a flaccid state, and easily yielding to impressions made on it by the fingers, while nothing can be ascertained, *per vaginam*, different from what may be perceived during the natural state of the abdomen. In short, in those unprofitable pains, the child's head may be sensibly elevated, since the uterine fibres are totally unconnected with the existing paroxysm. This translation of parturient energy may be effected either by direct, or by sympathetic excitement. The following is given as an elucidation.

"During the process of parturition, the urinary bladder becomes distended; the nature of the pain is now found to vary; the muscular fibres of the bladder are thrown into spasmodic action, and painful but imperfect feelings of a want to make water, during the paroxysms, take place; the uterine muscles are, at this time, to be found perfectly quiescent, however acute the paroxysm may be, the irritation of distention applied to the bladder having, by direct metastasis, acquired that energy which had previously actuated the uterus.

"Or it may happen that the irritation of the bladder, instead of producing increased actions of its own proper muscles, may excite sympathetic actions of a distant part, itself also remaining quiescent; these may be of the abdominal or other muscular parts, producing spasmodic affections of the same, or of the arterial system, giving rise to fever, syncope, &c. This is an instance of sympathetic metastasis." P. 66.

Under a full state of metastatic action, the progress of the labour is arrested, and this may continue for days, when occasionally the genuine uterine action is restored, and the child suddenly expelled.

The effect of metastatic determination of the parturient energy to other muscular parts is to throw those parts into inordinate or spasmodic action, accompanied by intense pain. In such cases it rarely happens that the pain ceases with the paroxysm, but, in bedside language, lingers in he parts affected, which are sore and tender to the touch. In the intervals of the metastatic paroxysm, the patient will often fall into an unrefreshing slumber, from which she awakes with starting or anxiety; or, she is harrassed with sickness or faintness In the metastatic state also, the mind is particularly depressed, and the patient forebodes a disastrous result. On the contrary, in the intervals of true uterine action, there is a freedom from distress, or soft and refreshing slumbers, the woman awakening to a renewal of her task with contented resignation.

Our author conceives that a metastatic translation of the parturient energy to the heart and arterial system, will cause feverish symptoms—and to the brain, puerperal convulsions; or the parturient energy may cease for a time in all parts.

In these unpropitious states of metastatic action, as was before observed, the progress of expulsion is, of course, arrested, and the change which has taken place will be readily ascertained, both by abdominal and vaginal examination. It is characteristic of the commencement of this metastatic state, that the pain accompanying the paroxysm is referred to a part which was not before affected; it will be found to occupy more frequently the abdominal or lumbar muscles, and occasionally those of the hips, thighs, bladder, and rectum. The nature of the pain also undergoes a material change; it becomes exceedingly acute; is described as grinding, cutting, or rending, producing every indication of extreme distress. The parts affected are not merely thrown into a spasmodic and painful state during the paroxysm, but continue, during the intervals of relaxation, to suffer considerable uneasiness; the pains being said to linger, and the parts affected remaining very sensible to the touch. The mental irritation and anxiety accompanying this state, operate an unfavourable influence upon the future progress of the case; being, in themselves, exciting causes of the metastatic state.

After this state of protraction has continued an indefinite time, it generally happens, that true uterine action will be suddenly restored, when the character of the case quickly changes again—the feelings of the patient are alleviated, the expulsive progress is resumed, and the termination often takes place with wonderful celerity; or the metastatic state may again recur, and that repeatedly.

The predisposition to the metastatic state seems to depend upon an increased susceptibility of the system to be acted upon by associations or sympathies, excited by bodily or mental stimuli; which susceptibility appears to derive its origin from the present state of human society, as influenced by the acquisition of habits originally unnatural, and by the peculiarities of its moral education or constitution.

But predisposition alone is not sufficient to give rise to metastatic action. There must be also exciting causes, which consist of *irritations,* applied in the parturient state, to some part of the uterine or general system, producing either a direct determination of the parturient energy to the seat of such irritation, or a sympathetic one of it to some distant part; thus giving rise to a counter and distinct action from what the uterus was previously experiencing

*Uterine* irritation may arise from irregular form of the uterine contents, as where there is a plurality of children, a monstrosity or disease, or an improper shape or position of the bones of the head. Here undue pressure on the uterus or its orifice, during parturition, may act as an inordinate irritation, and give rise to the metastatic state.

A premature evacuation of the liquor amnii, by rupture of the membranes, under an uncontracting or inactive state of the uterus, may, our author imagines, occasionally produce suspension of true uterine action, in consequence of the uterine parietes being left in a loose uncontracted state around their contents, by which the proper apposition of the presenting parts to the orifice is prevented.

When the uterine actions do re-commence, the metastatic state is apt to succeed, particularly if the os uteri is in a sensible state.

*Vaginal* irritations are generally determined by rigidity, stricture, or an inflammatory state of the passage from the uterus.

*Visceral* irritations. It is well known that a large proportion of the diseases to which the human system is liable, originate in a deranged state of the abdominal viscera; and it may be advanced as an axiom, that every visceral irritation which is capable of exciting a violent effect upon the general system, will also have a tendency to influence the uterine actions. The secretions and excretions, therefore, should be strictly attended to. Distention of the urinary bladder is a frequent source of metastatic action. This too ought to be avoided.

*Mental irritations.* The actions of the mind, as exciting causes of deranged parturition, might be made to involve a series of inquiries of the highest intricacy. The mind, like the body, is fatigued by its action, and requires repose; an excessive expenditure of nervous power, in producing bodily action, debilitates the mind; and *vice versa,* an excessive expenditure of the same upon the operations of the mind, wearies and fatigues the body.

The exciting passions, as joy, hope, &c. in moderation, are found to produce general increased action of the arterial system, and hence have a favourable influence on parturition. When in excess they are injurious, as are all the turbulent passions, as well as the depressing.

We now come to an important portion of the work, in which the management of metastatic determinations to different parts, is detailed, and on which we must dwell with some minuteness.

1. *Removal of Causes.* Some of these are beyond our power, as irregular pressure from the contents of the uterus, &c. Where the cause is *thickness of the membranes,* the rupture of them is to be effected. *Relaxation of the uterus,* from premature discharge of the liquor amnii, will be best relieved by friction upon the abdomen, or stimulation of the uterus itself. *Rigidity of the os uteri* must be met by topical emollient injections, and fomentations with the steam of warm water. *Disordered state of the stomach* should be prevented by prohibiting improper ingesta, and confining the parturient patient to tea, toast water, or lemonade for common drink. When the contents of the intestinal canal, by their acrimony or mechanical obstruction, are suspected of inducing the metastatic state, they should be removed by castor oil, or some gentle cathartic; and if the pain, griping, or flatus be urgent, an opiate may afford relief, particularly after the purging.

But the removal of the spasmodic affections, both by abstracting their causes, and by other means, is a most important part of the treatment. The internal antispasmodics, as æther, ammonia, wine, camphor, opium, venesection are, Mr. Power thinks, " to be used with distrust and circumspection." In respect to venesection, our author believes that its good effects are attributable to the diminished action, and consequent degree of supension which it produces, the metastatic affection thereby being allowed to cease, and the muscles which had been acting inordinately, to recover themselves, after which the subsequent renewal of action may prove of the true uterine kind. It is not to be hastily or precipitately prescribed, as it alarms the patient, and the mental emotion has an injurious effect.

The *external* antispasmodics, on which our author lays great stress, are embrocations, fomentations, warmth, pressure, and friction, to each of which we shall devote a few lines. The good effects of embrocations are dependent principally upon the warmth and friction conjoined with their use. Fomentations are of considerable importance, as, independent of their being powerful auxiliaries in the removal of spasm, they afford real comfort to the patient. They may be freely applied to any part of the external surface, and should be vigorously persisted in, by keeping up a regular succession of flannels to the parts affected. They are also locally serviceable in painful affections of the pubic region, pudenda, and perinæum, where other external means are not readily applicable. A few weeks ago we saw a curious instance of the effects of fomentations. A lady in the fifth month of pregnancy was harrassed for two days with periodical colicky pains about the umbilical region, returning every ten or fifteen minutes, but totally different, according to her own account, from labour pains, of which she had previous experience. Anodynes produced no effect on these pains, and there was no fever present. A fomentation was ordered to the abdomen, and in five minutes after the first wet flannel was applied, true labour pains came on, which the lady instantly recognized, and in half an hour she aborted.

*Injections* into the rectum will be of great and frequent advantage in the removal of the metastatic state, particularly when the spasms are referrible to the muscles around the rectum, coccyx, and lower part of the intestinal canal, or posterior portion of the bladder. Warm water alone may be used, or it may be rendered anodyne by the addition of from forty to one hundred drops of laudanum; or stimulant, by adding a table spoonful of common salt.

*Warmth* may be applied in a dry state by a warm hand, a bag of heated oats, or a bladder half filled with hot water.

*Pressure* often relieves spasm, and is exemplified by the anxiety which the patient feels to have her back supported during the paroxysm of pain.

"An excellent mode of adhibiting it will be found in the application of a napkin expanded about the abdomen, and tied tightly round the back, which may be tightened at pleasure."

*Friction*, though recommended as an adjuvant in hastening the expulsion of the placenta, has not hitherto been treated of for the relief of protracted parturition. Our author having been long in the habit of employing vigorous friction for the removal of spasm, was naturally induced to try it in the metastatic spasm of the parturient state, and the result exceeded his most sanguine expectations. The patient sometimes complains at first of the abdominal soreness, when the friction is applied, but this inconvenience speedily vanishes under its use. In general, when carefully applied, the relief and comfort experienced from it will both surprise and gratify her.

"In some, the improper action will be removed almost instantly, and as it were by a miracle; so that a case which had been protracted for the greater part of a week, under the most intense suffering, without the least progress, has been happily terminated in fifteen or twenty minutes from the commencement of the friction." P. 229.

It is not only during actual parturition that the use of friction is beneficial; it may also be employed, with decided advan'age, in precursory pains, and subsequently in the removal of after-pain. Friction is more particularly applicable to cases of metastasis affecting the external muscles, especially of the belly, back, hips, sacrum, &c. It is not so efficaciously applied with the palm or flat of the hand. The friction is not then so regular, the warmth and glow attending it are less excited, and the exertions of the operator are materially increased.

"The better mode of applying it is with the ends of the fingers, applied together, so as to form the segment of a circle, and moved over the part to be rubbed, in much the same way as the sound is elicited from a tambourine; this must, however, be done with great celerity, making from 130 to 150 motions of the hand in a minute, and at the same time with such degree of pressure as will produce considerable warmth and glowing feel of the part. The application should be made to the skin itself, and not through the medium of cloathing, and must be vigorously kept up in the above described manner, and extended with rapidity over the part affected; and if the spasmodic action should be found to vary its situation, it must instantly follow it." P. 232.

Our limits have already been passed, and we are unable to carry our analysis farther; especially on an elementary work. We conceive, that although the author is at times very prolix, yet that he evinces a considerable share of originality, with sound judgment, and acute observation. We believe the present analysis will show that there is a good deal of novelty in the views of the author, and that these views appear to lead directly to practical improvements in the management of parturition. This is no mean merit in these days. We need say no more in recommendation of the work.

